# Differences in the distribution of stroke subtypes in a UK black stroke population – final results from the South London Ethnicity and Stroke Study

**DOI:** 10.1186/s12916-016-0618-2

**Published:** 2016-05-20

**Authors:** Giosue Gulli, Loes C. A. Rutten-Jacobs, Lalit Kalra, Anthony G. Rudd, Charles D. A. Wolfe, Hugh S. Markus

**Affiliations:** Neurology, Frimley Park Hospital, Surrey, UK; Department of Clinical Neurosciences, Stroke Research group, University of Cambridge, Cambridge, UK; Department of Basic and Clinical Neurosciences, Institute of Psychiatry, Psychology and Neurosciences, King’s College London, London, UK; Division of Health and Social Care Research, King’s College London, London, UK; Stroke Unit, St. Thomas’ Hospital, London, UK; Guy’s and St. Thomas’ NHS Foundation Trust, National Institute for Health Research (NIHR) Biomedical Research Centre, London, UK

**Keywords:** Stroke, Risk factors, Ethnicity, Epidemiology

## Abstract

**Background:**

Stroke incidence is increased in Black individuals but the reasons for this are poorly understood. Exploring the differences in aetiological stroke subtypes, and the extent to which they are explained by conventional and novel risk factors, is an important step in elucidating the underlying mechanisms for this increased stroke risk.

**Methods:**

Between 1999 and 2010, 1200 black and 1200 white stroke patients were prospectively recruited from a contiguous geographical area in South London in the UK. The Trial of Org 10172 (TOAST) classification was used to classify stroke subtype. Age- and sex-adjusted comparisons of socio-demographics, traditional vascular risk factors and stroke subtypes were performed between black and white stroke patients and between Black Caribbean and Black African stroke patients using age-, sex-, and social deprivation-adjusted univariable and multivariable logistic regression analyses.

**Results:**

Black stroke patients were younger than white stroke patients (mean (SD) 65.1 (13.7) vs. 74.8 (13.7) years). There were significant differences in the distribution of stroke subtypes. Small vessel disease stroke was increased in black patients versus white patients (27 % vs. 12 %; OR, 2.74; 95 % CI, 2.19–3.44), whereas large vessel and cardioembolic stroke was less frequent in black patients (OR, 0.59; 95 % CI, 0.45–0.78 and OR, 0.61; 95 % CI, 0.50–0.74, respectively). These associations remained after controlling for traditional vascular risk factors and socio-demographics. Black Caribbean patients appeared to have an intermediate risk factor and stroke subtype profile between that found in Black African and white stroke patients. Cardioembolic stroke was more strongly associated with Black Caribbean ethnicity versus Black African ethnicity (OR, 1.48; 95 % CI, 1.04–2.10), whereas intracranial large vessel disease was less frequent in Black Caribbean patients versus Black African subjects (OR, 0.44; 95 % CI, 0.24–0.83).

**Conclusions:**

Clear differences exist in stroke subtype distribution between black and white stroke patients, with a marked increase in small vessel stroke. These could not be explained by differences in the assessed traditional risk factors. Possible explanations for these differences might include variations in genetic susceptibility, differing rates of control of vascular risk factors, or as yet undetermined environmental risk factors.

## Background

Stroke is the second leading cause of death worldwide and is a major cause of disability [1]. Stroke incidence is increased in European and US black individuals compared to white individuals [2, 3]; age-adjusted incidence has been estimated to be between 2.2 and 2.4 times higher compared with white individuals, with differences being particularly marked at younger ages [2]. The reasons for this increase remain incompletely explained. Adjustment for conventional risk factors attenuates this excess risk by approximately 40 %, with systolic blood pressure playing a major role [4]. Further adjustment for socioeconomic factors increases the mediation to 50 % [4]. However, this means that half of the excess risk seen was not attributable to traditional risk factors or socioeconomic factors, leading to the suggestion that non-traditional risk factors including genetic predisposition may be important [4, 5]. Stroke in black individuals remains a particularly important public health concern as temporal analyses in both the USA and UK suggest that, while stroke incidence is declining in white populations, a similar decline is not occurring in black populations [2, 6].

One striking finding, although derived from limited data, is a difference in the distribution of stroke subtypes between black and white stroke patients. It has been reported that small vessel disease is more common in black stroke patients [7–11]. Limited data also suggest intracranial stenosis may be more common in black stroke patients [11]. These findings suggest biological factors may play a role in the differing stroke incidence and distribution seen between different ethnic groups. A further understanding of these differences in stroke subtypes, and the extent to which they are explained by conventional and novel risk factors, is likely to be important in elucidating the mechanisms for increased stroke incidence found in black individuals. However, studies of stroke subtypes to date have been small and the levels of investigation have not always been sufficient to allow accurate subtyping based on a pathophysiological classification. Furthermore, it has also been suggested that risk factor profiles [3], and possibly the pattern of stroke subtypes [8], may differ between individuals of Black African and Black Caribbean ethnicity.

To address these issues and to investigate the mechanisms underlying the increased stroke incidence in black individuals, the South London Ethnicity and Stroke Study (SLESS) was established, recruiting 1200 consecutive black and 1200 white stroke admissions from a geographically contiguous area in London, UK. Interim results have been previously published on 600 patients per group [5]. Here, we present the final results on the complete sample with a particular focus on the distribution of stroke subtypes in the black patients, compared with a white stroke population from the same geographical area, and investigate the role of risk factors in explaining this difference. We also determined whether there were differences in stroke subtype profiles between black stroke patients of African and African Caribbean ethnicity.

## Methods

### Study population

The SLESS is a prospective study that recruited 1200 consecutive black patients with stroke from a contiguous geographical area in South London in the UK, covered by three acute hospitals (Guy’s and St Thomas’ Hospitals, King’s College Hospital, and St George’s Hospital). Recruitment occurred between 1999 and 2010. All hospitals have a specialised stroke unit and a rapid-access transient ischemic attack clinic. Ethnicity was defined according to the UK Census 2001 definition and classified as Black African or Black Caribbean [12]. Consecutive recruitment of 1200 white patients of European ancestry from the same geographical region who presented with stroke to St George’s Hospital was carried out over the period October 2003 to July 2009.

### Ethics, consent and permissions

The study protocol had ethics approval (Wandsworth Local Research Ethics Committee), and informed consent was obtained from all participants.

### Clinical assessment

All patients underwent standardized clinical assessment (Table 1). Risk factor information and other clinical and investigation details were prospectively collected on a standardized proforma. Hypertension was defined as systolic blood pressure ≥ 140 mm Hg or diastolic blood pressure ≥ 90 mm Hg persisting > 7 days after the acute event or pre-stroke treatment with antihypertensive drugs [13]. Diabetes mellitus was defined as a previous diagnosis of type I or II diabetes, or at least two random glucose readings of ≥ 11.1 mmol/L or fasting blood glucose readings of ≥ 7.0 mmol/L. Blood glucose readings used in diagnosis of diabetes were taken after the acute phase of stroke to exclude acute transient elevation of glucose as a stress response after stroke [14]. Hypercholesterolemia was defined as serum cholesterol > 5.2 mmol/L or pre-stroke treatment with a cholesterol-lowering agent [15]. A history of myocardial infarction, peripheral vascular disease, and atrial fibrillation was recorded from clinical history, notes review and family doctor referral letter. A positive smoking history was recorded in those who had smoked at any time in their lives. Weight and height were recorded and used to determine body mass index (BMI) in kg/m^2^. Socioeconomic factors were estimated using the Townsend deprivation index, which records values for all individual postcodes in England [16]. A higher index value corresponds to increased deprivation.

**Table 1:** Investigations to look at stroke pathophysiology performed in the different groups

	Black stroke patients			White stroke patients
	All	Caribbean	African	
	*n* = 1200 *n* (%)	*n* = 809 *n* (%)	*n* = 391 *n* (%)	*n* = 1200 *n* (%)
ECG	1183 (98.6)	795 (98.3)	388 (99.2)	1180 (98.3)
24 hr ECG	81 (6.8)	54 (6.7)	27 (6.9)	142 (11.8)
Echocardiogram	616 (51.3)	396 (48.9)	220 (56.3)	572 (47.7)
Carotid duplex^a^	989 (95.1)	687 (95.9)	302 (93.2)	1008 (92.9)
Brain CT	1143 (95.3)	772 (95.4)	371 (94.9)	1159 (95.4)
Brain MRI	573 (47.8)	375 (46.4)	198 (50.6)	526 (43.8)
DWI-MRI	454 (37.8)	300 (37.1)	154 (39.4)	396 (33.0)
CT-angiography	164 (13.7)	101 (12.5)	63 (16.1)	160 (13.3)
MR-angiography	349 (29.1)	225 (27.8)	124 (31.7)	295 (24.6)
Any extracranial vessel imaging^a^	996 (95.8)	690 (96.4)	306 (94.4)	1038 (95.6)
Thrombophilia screen^a^	214 (20.6)	107 (15.2)	107 (33.0)	145 (12.1)

ECG, Electrocardiography; CT, Computed tomography; MRI, Magnetic resonance imaging; DWI, Diffusion weighted imaging

All numbers represent the number of patients in whom the investigation has been done, with in brackets the corresponding percentage of all patients

^a^Percentages refer to ischaemic stroke patients only

### Stroke subtyping

One consultant neurologist (HSM) stroke subtyped all patients using data collected on a standard proforma with additional review of all original brain imaging in all patients, as well as review of original notes when necessary. Subtyping was not fully blinded to ethnicity because names were apparent on the brain imaging database system. The pathophysiological Trial of Org 10172 in Acute Stroke Treatment (TOAST) subtyping classification was used [17]. To avoid any bias resulting from different prevalence of risk factors, such as hypertension between the two groups, the presence of hypertension and diabetes was not used as a criterion in the diagnosis of subtypes; the original TOAST classification used it as an indicator of small vessel disease. In patients with previous stroke, subtyping was performing on the current stroke.

The degree of leukoaraiosis was recorded by review of original brain imaging using the semi-quantitative Fazekas scale [18] and the presence of confluent leukoaraiosis (grade ≥ 2) was recorded.

Where intracranial computed tomography angiography or magnetic resonance angiography had been performed the presence of intracranial stenosis was determined. The degree of intracranial stenosis was estimated using the WASID definition, where the numerator is the diameter of the artery at the site of the most severe stenosis and the denominator is the diameter of the normal proximal vessel diameter [19]. For an intracranial stenosis to be identified as the pathophysiological cause of the stroke, the stenosis had to be > 50 % and in the arterial territory of the stroke. However, we also performed a separate analysis of the prevalence of intracranial stenosis (>50 %) in any intracranial vessel, whether symptomatic or not.

### Statistical analysis

BMI was missing in 381 (31.8 %) and 90 (7.5 %) of white and black patients, respectively. White patients with missing BMI were more likely to be women, with higher age compared to the white patients in whom BMI was recorded. Other risk factors were not associated with missing BMI. On the other hand, hypertension, hyperlipidaemia and diabetes were strongly associated with BMI levels in those with BMI measurements. These missing data were handled using multiple imputation by the method of chained equations [20, 21], under the assumption that missing data was missing at random.

Five complete imputed datasets were created using predictive mean matching. All variables considered in the subsequent regression analyses, including hypertension, hyperlipidaemia and diabetes, were included in the imputation model. Regression analyses were performed on each of the imputed datasets individually and subsequently the coefficients were pooled using Rubin’s rules [22]. Restricting the analyses to only patients with complete data, yielded similar point estimates as obtained in the imputed datasets.

First, all of the following analyses were performed to compare black with white patients. Subsequently, black patients were grouped into Black African or Black Caribbean and all analyses were repeated comparing the two groups of black patients separately with white patients and comparing them with each other. The association of single risk factors and stroke subtypes with ethnicity was assessed using logistic regression analysis, adjusting for age and sex. Subsequently, multivariable logistic regression analysis was used to compare risk factor profiles between ethnic groups. All risk factors, independent of their statistical significance in the univariable analyses were entered in the multivariable model. Similarly, the association of stroke subtype with ethnicity was assessed in a multivariable model in which the associations were adjusted for risk factors and deprivation index. Atrial fibrillation was not included in the latter model because of its use as a criterion for diagnosing the cardioembolic stroke subtype. Sample size calculations performed showed that a sample size of 1200 black patients would allow an increase in intracranial stenosis from 4 % in African Caribbean stroke patients to 8 % in Black African stroke patients with a ratio of Black Caribbean to Black African of 2:1, alpha 0.05, and power 0.8. Statistical analyses were performed using the statistical software R version 3.2.1 (http://www.R-project.org).

## Results

### Demographic and risk factor differences between black and white stroke patients

Demographics and risk factors in black and white stroke patients are shown in Table 2. Black stroke patients were, on average, 10 years younger than white stroke patients (65.1 (SD, 13.7) years vs. 74.8 (SD, 13.7) years), were more likely to be male (59.7 % vs. 49.9 %, *P* < 0.001), and had higher levels of socioeconomic deprivation (*P* < 0.001). Among black stroke patients, Caribbean patients were on average 8 years older than African patients, less likely to be male (57.8 % vs. 63.4 %, *P* = 0.03) and had lower levels of socioeconomic deprivation (*P* < 0.001).

**Table 2:** Demographics and risk factors in white and black stroke populations

	White stroke patients (*n* = 1200) *n* (%)	Black stroke patients		
		All (*n* = 1200) *n* (%)	Caribbean (*n* = 809) *n* (%)	African (*n* = 391) *n* (%)
Age, mean years (SD)	74.8 (13.7)	65.1 (13.7)	67.7 (13.1)	59.6 (13.0)
Men, *n* (%)	599 (49.9)	716 (59.7)	468 (57.8)	248 (63.4)
Hypertension, *n* (%)	875 (72.9)	1007 (83.9)	682 (84.3)	325 (83.1)
Diabetes, *n* (%)	219 (18.3)	490 (40.8)	350 (43.3)	140 (35.8)
Hypercholesterolaemia, *n* (%)	733 (61.1)	673 (56.1)	461 (57.0)	212 (54.2)
Smoking, *n* (%)	742 (61.8)	485 (40.4)	388 (48.0)	97 (24.8)
Body mass index, kg/m^2^ mean (SD)^a^	25.0 (5.8)	27.4 (5.7)	27.3 (5.8)	27.4 (5.5)
Ischaemic heart disease, *n* (%)	270 (22.5)	149 (12.4)	112 (13.8)	37 (9.5)
Peripheral vascular disease, *n* (%)	112 (9.3)	51 (4.3)	40 (4.9)	11 (2.8)
Atrial fibrillation, *n* (%)	395 (32.9)	152 (12.7)	111 (13.7)	41 (10.5)
Townsend deprivation index, mean (SD)	2.8 (2.8)	6.6 (3.5)	6.3 (2.9)	7.1 (2.9)

^a^Body mass index was missing in 381 (31.8 %) and 90 (7.5 %) of white and black patients, respectively

Age- and sex-adjusted analyses showed that black patients were more likely to have hypertension, diabetes, increased BMI and increased levels of socioeconomic deprivation compared to white patients, but were less likely to have hypercholesterolaemia, ischaemic heart disease, peripheral vascular disease or atrial fibrillation and were less likely to be smoking (Table 3). All these differences for black patients compared to white patients remained for both Caribbean patients and African patients when they were compared separately with white patients. Among black stroke patients, age- and sex-adjusted analyses showed that Caribbean patients were less likely to have hypertension than African patients, had lower levels of socioeconomic deprivation, but were more likely to be smoking.

**Table 3:** Age- and sex-adjusted comparison of risk factors between white and black stroke populations

	Black vs. White		Black Caribbean vs. White		Black African vs. White		Black Caribbean vs. Black African	
	OR (95 % CI)	*P*	OR (95 % CI)	*P*	OR (95 % CI)	*P*	OR (95 % CI)	*P*
Age^a^	0.95 (0.94–0.96)	<0.001	0.96 (0.96–0.97)	<0.001	0.93 (0.92–0.94)	<0.001	1.05 (1.04–1.06)	<0.001
Male sex^b^	1.18 (0.99–1.40)	0.07	1.15 (1.15–1.38)	0.16	1.15 (0.88–1.50)	0.30	0.77 (0.60–1.00)	0.05
Hypertension	3.23 (2.55–4.08)	<0.001	2.97 (2.97–3.84)	<0.001	4.43 (3.04–6.45)	<0.001	0.63 (0.43–0.91)	0.02
Diabetes	3.55 (2.91–4.32)	<0.001	3.76 (3.76–4.64)	<0.001	3.06 (2.30–4.07)	<0.001	1.05 (0.81–1.37)	0.70
Hypercholesterolaemia	0.81 (0.68–0.97)	0.02	0.84 (0.84–1.01)	0.07	0.75 (0.58–0.98)	0.03	1.00 (0.77–1.28)	0.97
Smoking	0.31 (0.26–0.38)	<0.001	0.43 (0.43–0.52)	<0.001	0.14 (0.10–0.19)	<0.001	3.79 (2.81–5.11)	<0.001
Body mass index	1.06 (1.03–1.09)	<0.001	1.07 (1.07–1.09)	<0.001	1.06 (1.03–1.09)	<0.001	1.00 (0.98–1.03)	0.93
Ischaemic heart disease	0.62 (0.50–0.79)	<0.001	0.64 (0.64–0.82)	<0.001	0.55 (0.37–0.81)	0.003	1.09 (0.72–1.64)	0.69
Peripheral vascular disease	0.52 (0.36–0.73)	<0.001	0.56 (0.56–0.82)	0.003	0.39 (0.20–0.74)	0.004	1.34 (0.67–2.68)	0.41
Atrial fibrillation	0.45 (0.36–0.56)	<0.001	0.43 (0.43–0.55)	<0.001	0.48 (0.33–0.70)	<0.001	0.97 (0.65–1.46)	0.90
Townsend deprivation index	1.57 (1.51–1.64)	<0.001	1.59 (1.59–1.67)	<0.001	1.64 (1.54–1.75)	<0.001	0.92 (0.88–0.96)	<0.001

Odds ratios (OR) and *P* values for each risk factor were obtained using logistic regression analyses adjusted for age and sex

^a^Only adjusted for sex

^b^Only adjusted for age

CI, Confidence interval

In view of the strong relationship between risk factors and age, comparisons of risk factors between groups were performed in multivariable analyses controlling for all risk factors and social deprivation index. In these multivariable analyses, male sex, lower age, hypertension, diabetes, higher BMI, higher levels of socioeconomic deprivation, but lower prevalence of hypercholesterolaemia, smoking, ischaemic heart disease and atrial fibrillation were associated with black stroke patients versus white stroke patients (Table 4). Similar associations were shown for both Caribbean patients and African patients when they were compared separately with white patients. In comparison of Black Caribbean patients versus Black African patients, higher age and smoking were stronger risk factors for Black Caribbean patients, whereas male sex, hypertension and higher levels of socioeconomic deprivation were stronger risk factors for Black African patients.

**Table 4:** Multivariable comparison of risk factors between white and black stroke populations

	Black vs. White		Black Caribbean vs. White		Black African vs. White		Black Caribbean vs. Black African	
	OR (95 % CI)	*P*	OR (95 % CI)	*P*	OR (95 % CI)	*P*	OR (95 % CI)	*P*
Age	0.95 (0.94–0.96)	<0.001	0.96 (0.95–0.97)	<0.001	0.93 (0.92–0.95)	<0.001	1.05 (1.04–1.07)	<0.001
Male sex	1.81 (1.42–2.31)	<0.001	1.53 (1.18–1.98)	0.001	2.35 (1.59–3.47)	<0.001	0.50 (0.37–0.67)	<0.001
Hypertension	2.94 (2.16–4.00)	<0.001	2.55 (1.83–3.53)	<0.001	3.94 (2.39–6.47)	<0.001	0.59 (0.39–0.88)	0.01
Diabetes	2.54 (1.97–3.28)	<0.001	2.74 (2.10–3.58)	<0.001	1.91 (1.28–2.85)	0.002	1.10 (0.83–1.46)	0.50
Hypercholesterolaemia	0.66 (0.52–0.83)	<0.001	0.64 (0.50–0.83)	<0.001	0.75 (0.52–1.09)	0.13	1.04 (0.79–1.37)	0.76
Smoking	0.27 (0.21–0.35)	<0.001	0.36 (0.27–0.46)	<0.001	0.14 (0.09–0.20)	<0.001	3.98 (2.93–5.41)	<0.001
Body mass index	1.03 (1.00–1.07)	0.04	1.03 (1.01–1.06)	0.01	1.04 (1.01–1.08)	0.01	1.01 (0.99–1.04)	0.32
Ischaemic heart disease	0.73 (0.54–0.98)	0.04	0.72 (0.52–0.99)	0.05	0.68 (0.41–1.14)	0.14	0.96 (0.62–1.49)	0.87
Peripheral vascular disease	0.73 (0.47–1.14)	0.16	0.71 (0.44–1.15)	0.17	0.71 (0.31–1.61)	0.41	0.95 (0.46–1.96)	0.89
Atrial fibrillation	0.53 (0.39–0.70)	<0.001	0.54 (0.40–0.74)	<0.001	0.49 (0.30–0.81)	0.006	0.97 (0.63–1.48)	0.87
Townsend deprivation index	1.61 (1.53–1.68)	<0.001	1.61 (1.53–1.69)	<0.001	1.69 (1.57–1.82)	<0.001	0.91 (0.87–0.95)	<0.001

Odds ratios (OR) and *P* values for each risk factor were obtained using multivariable logistic regression analyses entering all variables in the model simultaneously

CI, Confidence interval

### Comparison of stroke subtypes

The distribution of stroke subtypes in the different groups is shown in Table 5.

**Table 5:** Stroke subtypes in white and black stroke populations

	Black stroke patients (*n* = 1200) *n* (%)	White stroke patients (*n* = 1200) *n* (%)	Black Caribbean stroke patients (*n* = 809) *n* (%)	Black African stroke patients (*n* = 391) *n* (%)
Intracerebral haemorrhage, *n* (%)	160 (13.3)	115 (9.6)	93 (11.5)	67 (17.1)
Large vessel disease, *n* (%)	109 (9.1)	159 (13.3)	69 (8.5)	40 (10.2)
Intracranial, *n* (%)	44 (3.7)	13 (1.1)	21 (2.6)	23 (5.9)
Extracranial, *n* (%)	65 (5.4)	146 (12.2)	48 (5.9)	17 (4.3)
Small vessel disease, *n* (%)	328 (27.3)	141 (11.8)	216 (26.7)	112 (28.6)
Cardioembolic, *n* (%)	220 (18.3)	390 (32.5)	167 (20.6)	53 (13.6)
Tandem, *n* (%)	43 (3.6)	68 (5.7)	29 (3.6)	14 (3.6)
Other cause, *n* (%)	57 (4.8)	48 (4.0)	39 (4.8)	18 (4.6)
Ischaemic unknown, *n* (%)	283 (23.6)	279 (23.3)	196 (24.2)	87 (22.3)

Age- and sex-adjusted analyses showed that small vessel disease stroke was more common in black stroke patients (Table 6). In contrast, large vessel stroke was more common in white stroke patients, and this was accounted for by an excess of extracranial large vessel stroke, whereas intracranial large artery stroke was more common in black stroke patients. Cardioembolic stroke was more common in white stroke patients.

**Table 6:** Age- and sex-adjusted comparison of stroke subtypes in white and black stroke populations

	Black vs. White		Black Caribbean vs. White		Black African vs. White		Black Caribbean vs. Black African	
	OR (95 % CI)	*P*	OR (95 % CI)	*P*	OR (95 % CI)	*P*	OR (95 % CI)	*P*
Intracerebral haemorrhage	1.18 (0.90–1.54)	0.25	1.15 (0.85–1.55)	0.36	1.34 (0.93–1.95)	0.12	0.80 (0.56–1.15)	0.23
Large vessel disease	0.59 (0.45–0.78)	<0.001	0.56 (0.42–0.77)	<0.001	0.69 (0.46–1.02)	0.06	0.76 (0.50–1.16)	0.21
Intracranial	2.99 (1.57–5.70)	<0.001	2.31 (1.13–4.71)	0.02	5.08 (2.38–10.83)	<0.001	0.44 (0.24–0.83)	0.01
Extracranial	0.39 (0.28–0.53)	<0.001	0.42 (0.30–0.59)	<0.001	0.31 (0.18–0.53)	<0.001	1.20 (0.67–2.14)	0.55
Small vessel disease	2.74 (2.19–3.44)	<0.001	2.65 (2.09–3.38)	<0.001	3.24 (2.37–4.44)	<0.001	0.86 (0.65–1.13)	0.27
Cardioembolic	0.61 (0.50–0.74)	<0.001	0.65 (0.52–0.81)	<0.001	0.47 (0.33–0.67)	<0.001	1.48 (1.04–2.10)	0.03
Tandem	0.76 (0.51–1.14)	0.19	0.73 (0.46–1.15)	0.18	0.92 (0.49–1.74)	0.81	0.87 (0.44–1.70)	0.68
Other cause	0.37 (0.23–0.58)	<0.001	0.46 (0.28–0.75)	0.002	0.21 (0.11–0.40)	<0.001	2.54 (1.35–4.76)	0.004
Ischaemic unknown	1.11 (0.91–1.35)	0.32	1.10 (0.89–1.37)	0.38	1.03 (0.76–1.40)	0.83	0.96 (0.71–1.29)	0.78

Odds ratios (OR) and *P* values for each stroke subtype were obtained using logistic regression analyses adjusted for age and sex

CI, Confidence interval

Differences in the distribution of stroke subtypes in black stroke patients versus white stroke patients was similar for both Caribbean patients and African patients when they were compared separately with white patients. Among black stroke patients, the cardioembolic stroke and other defined stroke subtypes were more strongly associated with Black Caribbean patients versus Black African patients, whereas intracranial large vessel disease was more strongly associated with Black African patients.

Controlling for all risk factors and social deprivation index did not change the distribution of differences in stroke subtypes between black patients and white patients (Table 7).

**Table 7:** Risk factor and deprivation adjusted comparison of stroke subtypes in white and black stroke populations

	Black vs. White		Black Caribbean vs. White		Black African vs. White		Black Caribbean vs. Black African	
	OR (95 % CI)	*P*	OR (95 % CI)	*P*	OR (95 % CI)	*P*	OR (95 % CI)	*P*
Intracerebral haemorrhage	1.03 (0.72–1.48)	0.86	1.02 (0.69–1.50)	0.93	1.16 (0.67–2.01)	0.61	0.99 (0.67–1.47)	0.96
Large vessel disease	0.52 (0.37–0.74)	<0.001	0.47 (0.32–0.70)	<0.001	0.65 (0.38–1.10)	0.11	0.67 (0.42–1.04)	0.08
Intracranial	1.80 (0.81–4.02)	0.15	1.61 (0.66–3.95)	0.30	3.27 (1.17–9.12)	0.02	0.46 (0.24–0.89)	0.02
Extracranial	0.39 (0.26–0.58)	<0.001	0.37 (0.24–0.57)	<0.001	0.35 (0.18–0.68)	0.002	0.93 (0.51–1.73)	0.83
Small vessel disease	2.60 (1.94–3.48)	<0.001	2.40 (1.76–3.28)	<0.001	3.61 (2.32–5.63)	<0.001	0.82 (0.61–1.11)	0.20
Cardioembolic	0.77 (0.59–1.00)	0.05	0.84 (0.63–1.11)	0.21	0.62 (0.39–0.97)	0.04	1.44 (0.99–2.10)	0.06
Tandem	0.83 (0.49–1.44)	0.51	0.91 (0.51–1.62)	0.74	0.66 (0.28–1.55)	0.35	0.89 (0.44–1.77)	0.73
Other cause	0.51 (0.29–0.90)	0.02	0.62 (0.34–1.14)	0.13	0.26 (0.11–0.61)	0.002	2.28 (1.19–4.38)	0.01
Ischaemic unknown	0.98 (0.76–1.28)	0.90	0.97 (0.73–1.28)	0.82	0.93 (0.62–1.40)	0.73	0.96 (0.70–1.32)	0.81

Odds ratios (OR) and *P* values for each stroke subtype were obtained using logistic regression analyses adjusted for age, sex, hypertension, diabetes, hypercholesterolaemia, smoking, body mass index, ischaemic heart disease, peripheral vascular disease and deprivation index

CI, Confidence interval

The distribution of other defined causes is shown in Table 8. The percentage of patients with other defined causes was similar among groups (4.0 % in white patients and 4.8 % in black patients). Patients with another defined cause were significantly younger than those with the other stroke subtypes (52.1 (SD, 17.1) years vs. 75.7 (SD, 12.7) years in white patients and 48.0 (SD, 14.0) vs. 65.9 (SD, 13.1) years in black patients). The most common other determined cause was extracranial cranial artery dissection, which had a similar frequency in both ethnic groups; 22 black patients and 25 white stroke patients. There were seven patients of stroke due to sickle cell disease only in the black stroke patients, and coagulopathy was more common in the black stroke patients (10 vs. 2 patients).

**Table 8:** Breakdown of other specified causes of stroke in the different groups

	Black	White
CADASIL	1	0
MELAS	1	0
Drug related	1	1
Endocarditis	0	2
HIV	3	1
Vasculitis	4	1
Cervical artery dissection	22	25
Sickle cell disease	7	0
Coagulopathy	10	2
Cancer	2	8
Iatrogenic	6	8
Total number of patients	57	48

CADASIL, Cerebral Autosomal-Dominant Arteriopathy with Subcortical Infarcts and Leukoencephalopathy; MELAS, Mitochondrial encephalomyopathy, lactic acidosis, and stroke-like episodes; HIV, Human immunodeficiency virus

To further explore differences in intracranial disease between different groups, all patients in which intracranial imaging was performed (25.8 % of white stroke patients, 40.5 % of black stroke patients) were reviewed. The presence of intracranial stenosis was increased in black compared with white stroke patients (98/486, 20.1 % vs. 37/310, 11.9 %, *P* < 0.0001). There was a trend towards increased intracranial stenosis in African compared with Caribbean stroke patients (24.7 % vs. 17.4 %, *P* = 0.054).

### Comparison of leukoaraiosis on neuroimaging

To further explore differences in the prevalence of small vessel disease in the different groups, we compared the presence of confluent leukoaraiosis on brain imaging. Due to the marked age dependence of leukoaraiosis, all analyses were age and sex adjusted. Confluent leukoaraiosis was more common in black compared with white stroke patients (odds ratio (OR), 1.61 (95 % confidence intervals (CI), 1.33–1.96), *P* < 0.001), and this difference persisted after controlling for all cardiovascular risk and socioeconomic deprivation (OR, 1.61 (95 % CI, 1.25–2.08), *P* < 0.001). Confluent leukoaraiosis was more common in Black African versus Black Caribbean stroke patients; age and gender adjusted (OR, 1.68 (95 % CI, 1.27–2.23), *P* < 0.001); and after adjustment for all risk factors and deprivation index (OR, 1.71 (95 % CI, 1.26–2.31), *P* < 0.001).

## Discussion

In this prospective study with recruitment from a contiguous geographical area of South London, UK, we found significant differences in the distribution of stroke subtypes between black and white stroke patients, with an increase in small vessel disease stroke, and a reduction in large artery and cardioembolic stroke. These differences persisted after controlling for traditional cardiovascular risk factors and degree of social deprivation.

A major finding from the SLESS study is the increase in small vessel disease seen in the black stroke patients. Small vessel stroke was 2.6 times more common in black patients, after controlling for risk factors and deprivation. Consistent with this, leukoaraiosis on brain imaging, which is a radiological marker of small vessel disease, was increased in black patients after controlling for risk factors and deprivation. An increase in small vessel disease in black stroke patients has been reported in some, but not all, previous studies comparing stroke subtypes between the two ethnic groups [7–11]. A major strength of our study was the degree of investigation of patients, which allowed detailed subtyping. This is particularly important for the small vessel disease subtype for which diagnosis, based on clinical syndrome alone using classifications such as the Oxfordshire Community Stroke Project Classification [23], can be inaccurate and may differentially affect prevalence rates of the small vessel disease subtype in different ethnic groups. In the interim SLESS analysis, we demonstrated that stroke subtyping using the clinical Oxfordshire Community Stroke Project Classification resulted in a higher frequency of small vessel disease particularly in the white patients, and an underestimation, compared with results based on the pathophysiological TOAST classification, of the OR of small vessel disease stroke in black versus white stroke patients (OR, 1.95 vs. 2.93) [5]. This difference was primarily accounted for by misdiagnosis of white stroke patients with a clinical lacunar syndrome and carotid stenosis as small vessel disease. Epidemiological studies which do not perform extracranial imaging in all patients will therefore underestimate the difference in small vessel disease between the two ethnic groups. In SLESS, 95 % of all ischemic strokes had this imaging and there was no difference in its use in the two ethnic groups. The increased small vessel disease we found in the black stroke patients is consistent with population-based studies looking at subclinical markers of small vessel disease, namely small deep infarcts and white matter hyperintensities. These have been shown to be increased in black individuals in both the US and UK [24, 25].

The reason for the increased small vessel disease seen in black populations is uncertain. Hypertension and diabetes are major risk factors for small vessel disease stroke and, in the present study, both were more common in the black stroke patients, but the increase in small vessel disease persisted after controlling for these risk factors [5]. It is also possible that increased severity of hypertension in the black patients could contribute to the increased risk of small vessel disease. There was a suggestion that the severity of hypertension was greater in black patients, with a trend for an increased number of antihypertensive agents before stroke. However, a recent analysis from the REGARDS study has suggested an alternative explanation – that there are ethnic differences in the impact of elevated blood pressure on stroke risk with similar levels leading to increased risk in black, compared with white, individuals [26]. The basis for such differences, and whether it represents biological or genetic differences or possibly residual confounding, is unknown.

Although large artery stroke was more common in white stroke patients, this was accounted for by a marked increase in stroke due to extracranial large artery stenosis, which was 2.6 times more common, on fully adjusted analysis. In contrast, stroke secondary to intracranial stenosis appeared to be more common in black stroke patients, with a 70 % increase although this did not reach significance on adjustment for risk factors and deprivation index. Consistent with this, the presence of any intracranial stenosis on intracranial angiography was more common in black stroke patients.

Cardioembolic stroke was more common in white stroke patients. This is likely due to the lower incidence of atrial fibrillation in black individuals, which has been the subject of recent interest. This observation has been reported in both classic epidemiological studies [27], and more recently in studies using implantable cardiac devices [28]. The reasons for this difference remain uncertain, but it was not explained by classical atrial fibrillation risk factors, which were more common in black individuals despite the reduction seen in atrial fibrillation prevalence [27].

The percentage of other defined causes was similar among black and white patients (4–5 %). However, age- and sex-adjusted analyses showed that this subtype was associated with white patients versus black patients and the prevalence of other defined causes among those younger than 50 years was almost double in white patients versus black patients. A likely explanation for this is the higher prevalence of classical vascular risk factors like hypertension and diabetes in the black population at younger ages than the white population, leading to more classical strokes at a younger age in black stroke patients versus white stroke patients [4].

The prevalence of diabetes was substantially higher in black compared to white patients (43 % in Black Caribbean patients, 36 % in Black African patients and 18 % in white patients). Further, in the multivariable analyses, diabetes was strongly associated with black versus white patients (no difference between Black Caribbean and Black African), independently of other demographic or vascular risk factors. Possible explanations for this difference might include differing rates of control of diabetes and other risk factors, undetermined environmental risk factors or differences in genetic susceptibility.

We also found differences in risk factor profiles and stroke subtypes between Black African and Black Caribbean stroke patients. The former are primarily first generation immigrants from Africa, while the latter are primarily first generation immigrants from the Caribbean islands. Black African patients were younger, more likely to be men, hypertensive, had higher deprivation index and were less likely to be smokers. While small vessel disease stroke was increased to a similar degree in both Black African and Caribbean individuals, intracranial large vessel disease was more common in Black African compared with Black Caribbean stroke patients, and this difference remained after controlling for risk factors. Cardioembolic stroke was less associated with the Black African group but this was not significant after adjustment for risk factors.

There are similarities, but some differences, between the pattern of stroke subtypes seen in this UK population and inner city US populations. While the increase seen in small vessel disease and intracranial large vessel disease is common across all studies, the US studies have shown equivalent or higher levels of extracranial large vessel disease in blacks and similar levels of cardioembolic stroke [10, 11]. Prevalence of hypertension, diabetes and hypercholesterolemia are similar amongst the two populations although the US populations were slightly older [11].

The mechanisms underlying the difference in stroke subtypes between the ethnic groups remain uncertain. One possibility is that genetic or other factors modulate the way in which conventional risk factors, such as hypertension, result in end organ damage. However, although we controlled for all risk factors and also a measure of social deprivation, it is also possible that there remain unadjusted differences in risk factor profiles that contribute to the differences in stroke subtypes. In addition, rates of suboptimal control of vascular risk factors might differ between groups. The stroke subtype profile in Black Caribbean patients appeared in some ways intermediate between that found in Black African and white stroke patients. Whether this reflects different risk factor exposure, with African patients having been exposed to a “western” risk factor environment for shorter durations or differing genetic admixture is unknown. The technique of genetic admixture data using genome wide association data may help resolve the relative role of genetic versus environmental influences [29].

Our study has a number of strengths. It included a large number of well-phenotyped black patients with stroke and had a much larger number of black strokes than previous studies looking at stroke subtypes. All patients were prospectively recruited and all stroke subtyping was performed by the same individual with review of original brain imaging. There was a high rate of investigation for causes of stroke, with all patients having brain imaging and 96 % having either carotid duplex or magnetic resonance angiography to image the extracranial vessels.

A potential limitation is that SLESS was not truly population based. However, the catchment areas of the three hospitals covered a contiguous geographical area. Patients were recruited not only from hospital admissions but also from outpatient stroke services. The community-based South London Stroke Register is nested within the geographical catchment area of SLESS, which allowed us to determine the proportion of our black and white patients with stroke in the community who were admitted to hospital. A detailed analysis of the first 600 patients found the proportion of both black and white patients admitted was very similar to that found in the South London Stroke Register population over the same period, suggesting at most a very small case ascertainment bias in our study population [5].

Moreover, the determination of self-reported ethnicity using the UK Census 2001 definition has limitations. Although patients reported of mixed-ethnicity were not included, there is still the possibility of admixture. In addition, ethnicity does not only represent genetic background, but also cultural and behavioural differences which can evolve within individuals over time and between generations [30], all of which are important in the risk of vascular disease.

Black patients were recruited from three acute hospitals within a contiguous geographical area in South London, whereas the white patients were only recruited in one of these three hospitals. This possibly might have induced bias in the comparison of black patients with white patients. However, we performed a sensitivity analysis, comparing stroke subtypes between black and white patients, in which we only included the black patients that were recruited from the same hospital as the white patients. The results of this analysis were similar to the full analysis and did not alter our conclusions. Therefore, we think that, if there would be any bias due to different inclusion sites, the effect would only have been small.

Another possible limitation is the slightly longer time period over which black stroke patients were recruited compared to white stroke patients. This might possibly have induced bias due to, for example, changing diagnostic techniques and treatment protocols. We performed a sensitivity analysis in which we repeated all analyses, only including the black stroke patients recruited from 2003 (75 % of all black patients). The sensitivity analyses did not show any changes in results compared with the original analysis.

## Conclusions

We demonstrated, in this large well-characterised stroke population, clear differences in the distribution of stroke subtypes between white and black stroke patients which could not be explained by differences in risk factor profiles. Black Caribbean patients appeared to have an intermediate risk factor and stroke subtype profile between that found in Black African and white stroke patients. The mechanism for the increase of small-vessel disease in black patients and large-artery disease in white patients is currently unknown. Further studies that include genetics and information on control of vascular risk factors are needed to explain these ethnicity-specific differences.

## Availability of data and materials

These data are not publicly available as these concern patient-related data.
